# Knowledge, Attitude, and Practice Regarding Digital Dental Technologies Among Dentists in Jiangsu Province

**DOI:** 10.3390/healthcare13030234

**Published:** 2025-01-24

**Authors:** Feng Zhu, Hong Yu, Zitao Wang, Xiaoxin Lu, Xiangfeng Meng, Rongrong Nie

**Affiliations:** 1 Department of Oral and Maxillofacial Surgery, Nanjing Stomatological Hospital, Medical School of Nanjing University, Nanjing 210008, China; drzf@nju.edu.cn; 2 Medical Department, Nanjing Stomatological Hospital, Medical School of Nanjing University, Nanjing 210008, China; skqzlb@163.com; 3 Department of Prosthodontics, Nanjing Stomatological Hospital, Medical School of Nanjing University, Nanjing 210008, China; xiaoliyh@126.com (H.Y.); lzjzwzt@yeah.net (Z.W.); 4 Department of Prosthodontic Technology, Nanjing Stomatological Hospital, Medical School of Nanjing University, Nanjing 210008, China; 5 Department of Geriatric Stomatology, Nanjing Stomatological Hospital, Medical School of Nanjing University, Nanjing 210008, China

**Keywords:** digital dental technology, dentistry, knowledge, attitude, and practice, cross-sectional study

## Abstract

**Objectives**: The aim of this study was to explore the knowledge, attitude, and practice (KAP) with regard to digital dental technologies among dentists in Jiangsu province. **Methods**: This web-based cross-sectional study enrolled dentists in Jiangsu province during June, 2023. A web-based questionnaire was used to collect the demographic characteristics of the participants and KAP regarding digital dental technologies. The structural equation model (SEM) was used to analyze the associations between KAP dimensions. **Results**: A total of 284 questionnaires were collected, of which 212 (74.65%) were valid, and 108 (50.94%) of the participants were male. Their knowledge, attitude, and practice scores were 14.33 ± 3.59 (possible range: 0–19), 26.17 ± 3.40 (possible range: 8–40), and 10.73 ± 3.11 (possible range: 4–16), respectively, indicating sufficient knowledge, a positive attitude, and proactive practice. Cone–beam computed tomography (CBCT) was the most well-understood digital dental technology among the dentists (205, 96.70%), followed by intraoral scanning/models and digital impression scanning/facial scanning (198, 93.40%), computer-aided design/computer-aided manufacture (CAD/CAM) (177, 83.49%), three-dimensional printing (175, 82.55%), and surgical guides (175, 82.55%). CBCT was the most frequently used technique (90.09%), followed by intraoral scanning (72.17%), impression scanning (54.25%), and CAD/CAM (42.45%). The SEM showed that knowledge had a direct effect on attitude (β = 0.283, *p* < 0.001), and attitude had a direct effect on practice (β = 0.280, *p* < 0.001), demonstrating that knowledge had an indirect effect on practice. Knowledge also had a direct effect on practice (β = 0.310, *p* < 0.001). **Conclusions**: Dentists in Jiangsu province had sufficient knowledge, a positive attitude, and proactive practice with regard to digital dental technologies. However, policies should encourage dentists to integrate digital technologies into their practice by establishing sufficient understanding and positive attitudes.

## 1. Introduction

Digital dental technology is a broad term that embraces multiple technological modalities and computer-based algorithms for the communication, documentation, planning, and delivery of dental therapy, having a great impact on dentistry and how dentists diagnose, plan treatments, and provide patient care [1,2]. Techniques including cone–beam computed tomography (CBCT) [3], intraoral scanning [4], digital impressions [5], custom computer-aided design/computer-aided manufacture (CAD/CAM) [6], and three-dimensional (3D) printing [7] are gaining acceptance and popularity in orthodontics, prosthodontics, and implant dentistry. Nowadays, CBCT is applied in almost all disciplines of oral medicine [3]. Intraoral scanning can reduce procedure time and improve patient comfort in fixed prosthodontics and implant dentistry [8]. The CAD/CAM system has greatly contributed to improving and simplifying diagnosis, treatment planning, and execution in orthodontics [9]. With recent advances in 3D imaging, computer-assisted surgical planning is now routinely used for analyzing craniofacial structures and improving outcome prediction in orthognathic surgery [10]. The integration of virtual customized bracket and 3D surgical planning using CAD/CAM can drastically shorten the treatment time [11]. In modern implantology, the application of surgical navigation systems is also becoming increasingly important [12]. Surgical guides are vital in assisting with the positioning of preoperatively virtually planned implants [13]. Medical robotics can enhance the quality of dental care and support human resources [14]. The continuous development of digital dental technologies ensures new opportunities in dentistry [15]. Therefore, a dentist’s understanding and mastery of digital technology is necessary.

In the past decade, China has developed policies to promote oral health and increased its investment in the oral industry, which has accelerated the application of digital dental technologies [16]. However, there are limited data about the actual use of digital dental technologies in China. In addition, the application of digital dental technologies may be affected by various factors, such as the preference and knowledge of dentists, but no studies have explored these factors. Knowledge, attitude, and practice (KAP) is a behavioral intervention theory, one of the common models used to explain how knowledge affects behaviors. In the KAP theoretical model, changes in human behavior can be divided into three continuous processes, acquiring knowledge, producing beliefs, and forming behaviors, and the most common approach in KAP assessment is the use of a questionnaire [17,18]. Previous studies on digital dental technologies generally focus on their technical properties or clinical performance [4,19]. Research on the KAP of dentists with regard to digital dental technologies is scarce, and most includes outdated data or evaluate a single KAP dimension without exploring the relationships between KAP dimensions [20,21]. To plan for the future acceptance and implementation of digital technology in dentistry, it is necessary to understand KAP with regard to digital dental technologies among dentists.

Located in eastern China, Jiangsu Province ranks among the top provinces in terms of gross domestic product (GDP) and per capita GDP and provides relatively advanced medical service capacity, and is hence a typical representative region for the application of digital dental technologies in China [22]. Therefore, this study aimed to explore KAP regarding digital dental technologies and their application among dentists in Jiangsu province. The influencing factors of KAP were explored and the following hypotheses were made based on KAP theory. H1: The participants with higher knowledge scores are more likely to have higher attitude scores. H2: The participants with higher knowledge scores are more likely to have higher practice scores. H3: The participants with higher attitude scores are more likely to have higher practice scores. This study could help discover the KAP gaps in digital dental technology among dentists, providing a basis for future research in related fields and informing policymakers so that they can implement targeted training and optimize policies in China and other developing countries.

## 2. Materials and Methods

### 2.1. Study Design and Participants

This web-based cross-sectional study enrolled dentists in Jiangsu province during June, 2023. The inclusion criteria included (1) registered dentists and (2) voluntary participation. The exclusion criteria included questionnaires with incomplete or conflicting responses. A self-administered questionnaire was distributed to the dentists through a convenience sampling method to collect their demographic information and KAP regarding digital dental technologies. The link to the questionnaire was distributed through the Jiangsu Dental Association. The minimum sample size required for this study was estimated to be 5–10 times the number of items in the KAP dimension [23], and thus, the minimum number of valid questionnaires was 165 (5 times the 33 questions about KAP) to analyze KAP. To heighten the representativeness of the sample, up to 10 dentists were included from the same medical center.

This study was ethically approved by the Institutional Review Board of our hospital (No. NJSH-2023NL-056; Date: 25 May 2023), and informed consent was obtained from all participants.

### 2.2. Procedures

The questionnaire was designed based on a previous study [24], and was modified by 2 senior experts. A pilot study was conducted among 45 dentists, and the Cronbach’s α was 0.782, which indicates good internal consistency.

The final questionnaire contained 4 dimensions: the demographic characteristics dimension, the knowledge dimension, the attitude dimension, and the practice dimension. The demographic characteristics dimension included gender, age, professional title, ethnicity, education, subdiscipline, working institutions, and years of work experience. The knowledge dimension consisted of 8 questions, of which Question 1, “Did you heard of digital dental technologies”, and Question 8, “4 + 6 = 13”, were used to verify the questionnaire’s validity. Questions 2 and 3 were scored by assigning 1 point for the option “Understand”, and Questions 4–7 were scored by assigning 1 point for the option “Correct”. The total scores of the knowledge dimension ranged from 0 to 19 points. The attitude dimension consisted of 8 questions using a 5-point Likert scale, with Questions 1–7 using forward scoring (0–4) and Question 8 using reverse scoring (4–0). The total scores of the attitude dimension ranged from 0 to 32. The practice dimension contained 5 questions, with Questions 1–4 using forward scoring (0–4), and the total scores of the practice dimension ranged from 0 to 16. A knowledge, attitude, and practice score > 60% of the total score in each dimension was considered to indicate “sufficient knowledge”, “positive attitude”, and “proactive practice” regarding digital dental technologies [25].

For quality control, the research team checked the answers of the collected questionnaires for validity. Questionnaires in which participants stated that they had never heard of digital dental technologies in Question 1 or replied “Correct” or “Unclear” to Question 8, “4 + 6 = 13”, in the knowledge dimension were considered to be invalid.

### 2.3. Statistical Analysis

Stata 17.0 (Stata Corporation, College Station, TX, USA) and AMOS 24.0 (IBM, Armonk, NY, USA) were used for the statistical analysis. Continuous data that conformed to a normal distribution are presented as the mean ± standard deviation (SD), and were compared using an independent-samples *t*-test or one-way analysis of variance (ANOVA). The categorical data are expressed as n (%). Post hoc pairwise comparisons were performed with post hoc Bonferroni tests. Pearson’s correlation analysis was used to analyze the correlation between the knowledge, attitude, and practice scores. A structural equation model (SEM) was used to analyze the correlations among KAP regarding digital dental technologies. A confirmatory factor analysis was conducted and the model fitting was evaluated with a model chi-square χ^2^ (CMIN)/degree-of-freedom (DF) test and the root-mean-square error of approximation (RMSEA), incremental fit index (IFI), Tucker–Lewis index (TLI), and comparative fit index (CFI). A two-sided *p* < 0.05 was considered statistically significant.

## 3. Results

A total of 284 questionnaires were collected, of which 1 was excluded as the participant had never heard of digital dental technologies and 71 were excluded for incorrect responses to Question 8, and 212 valid questionnaires were collected (effective rate: 74.65%). Of the participants, 108 (50.94%) were male, 116 (54.72%) were 40 years old or below, 89 (41.98%) had a professional title of senior, and 150 (70.75%) had more than 10 years of work experience. The confirmatory factor analysis is shown in Supplementary Figure S1, and the fitting index of the SEM (CMIN/DF = 1.910, RMSEA = 0.066, IFI = 0.828, TLI = 0.811, CFI = 0.825) showed that the model had good construct reliability (Supplementary Table S1). The knowledge, attitude, and practice scores regarding digital dental technology were 14.33 ± 3.59, 26.17 ± 3.40, and 10.73 ± 3.11, respectively, indicating sufficient knowledge, a positive attitude, and proactive practice. There were significant differences in the knowledge scores among participants with different genders, professional titles, education, grades of working institutions, and years of work experience (all *p* < 0.05); significant differences in the attitude scores among participants with different professional titles (*p* = 0.009); and significant differences in the practice scores among participants with different genders, ages, professional titles, education, and years of work experience (all *p* < 0.05) (Table 1).

**Figure 1:**
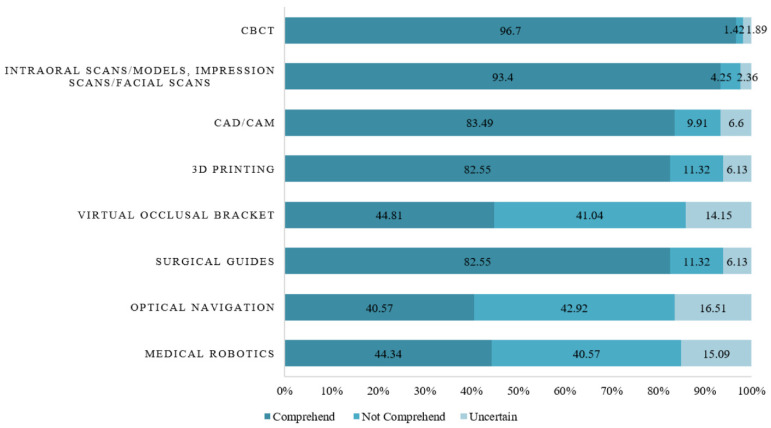
Comprehension of different digital dental technologies. CBCT: cone–beam computed tomography; CAD: computer-aided design; CAM: computer-aided manufacture; 3D: three-dimensional.

Dentists had a good understanding of the application of digital dental technology in oral implantology (197, 92.92%), prosthodontics (194, 91.51%), the diagnosis of dental disease (185, 87.26%), orthodontics (177, 83.49%), endodontics (156, 73.56%), and maxillofacial surgery (138, 65.09%), while a mere of 95 (44.81%) participants were aware of the application of digital technology in periodontics (Appendix A Table A1). CBCT was the most well understood digital dental technology among the dentists (205, 96.70%), followed by intraoral scanning/models and digital impression scanning/facial scanning (198, 93.40%), CAD/CAM (177, 83.49%), 3D printing (175, 82.55%), and surgical guides (175, 82.55%). Less than half of the dentists were aware of the application of virtual occlusal brackets (95, 44.81%), medical robotics (94, 44.34%), and optical navigation (86, 40.57%) (Figure 1). A total of 207 (97.64%) dentists believed that “the data of digital technologies is easy to share/store/transfer”, while only 144 (67.92%) dentists thought “digitally designed and produced restorations give better outcomes than with traditional methods” (Appendix A Table A1). It is evident that dentists in China had overall sufficient knowledge, but there were still gaps in the application of digital technology in periodontics, as well as the application of virtual occlusal brackets, medical robotics, and optical navigation.

The surveyed dentists favored the applications of digital technologies and showed positive attitudes towards their development. Most participants agreed or strongly agreed with the advantages of digital technologies in improving efficiency (197, 92.92%) and enhancing the consultation experience of dental patients (201, 94.81%), the developing trend of using digital technologies in dentistry (208, 98.11%), the significant role of professional dentists in the application of digital technologies (194, 91.51%), and the need to add digital technologies to the curriculum of dental students (196, 92.45%). Nearly half of the participants (127, 59.91%) agreed or strongly agreed with the irreplaceable role of digital technologies in dentistry, while 58 (27.36%) participants held a neutral attitude towards it. A total of 45 (21.23%) of participants were concerned that they might be replaced by digital technologies in the future, while other participants showed a neutral attitude (80, 37.74%) or disagreed/strongly disagreed (87, 41.04%) with this (Appendix A Table A2).

**Figure 2:**
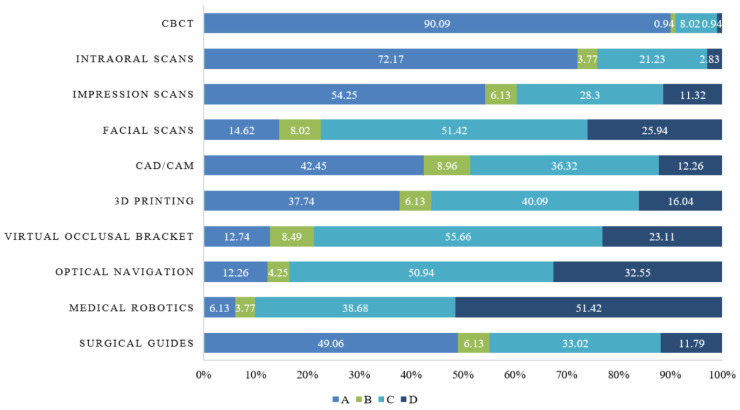
Application of different digital dental technologies. CBCT: cone–beam computed tomography; CAD: computer-aided design; CAM: computer-aided manufacture; 3D: three-dimensional. A: already in application; B: the hospital is equipped with relevant devices, but the technique has not been applied; C: the hospital is not equipped with relevant devices now but is willing to equip and apply the technique; D: the hospital is not equipped with relevant devices now and sees no need for its application.

As for practice, dentists had overall proactive practices regarding digital technologies. A total of 55 (25.94%) participants claimed that they always introduced the use of digital technologies to patients, while 47 (22.17%) always recommended that patients undergo digital imaging, 37 (17.45%) always used digital technologies to assist in the treatment of dental diseases, and 36 (16.98%) always proactively learned about advances in the application of digital technologies (Appendix A Table A3). As for the application of digital dental technology, CBCT was the most frequently used technique (90.09%), followed by intraoral scanning (72.17%), impression scanning (54.25%), and CAD/CAM (42.45%). However, some hospitals are not equipped with relevant devices now but are willing to equip and apply these techniques. This unmet need for digital application was found in virtual occlusal brackets (55.66%), optical navigation (50.94%), 3D printing (40.09%), medical robotics (38.68%), and surgical guides (33.02%). In addition, 51.42%, 32.55%, 25.94%, and 23.11% of the dentists saw no need for the current application of medical robotics, optical navigation, facial scanning, and virtual occlusal brackets, respectively, in their working institutions (Figure 2), indicating the relatively slow development and lack of applications of these digital technologies.

**Figure 3:**
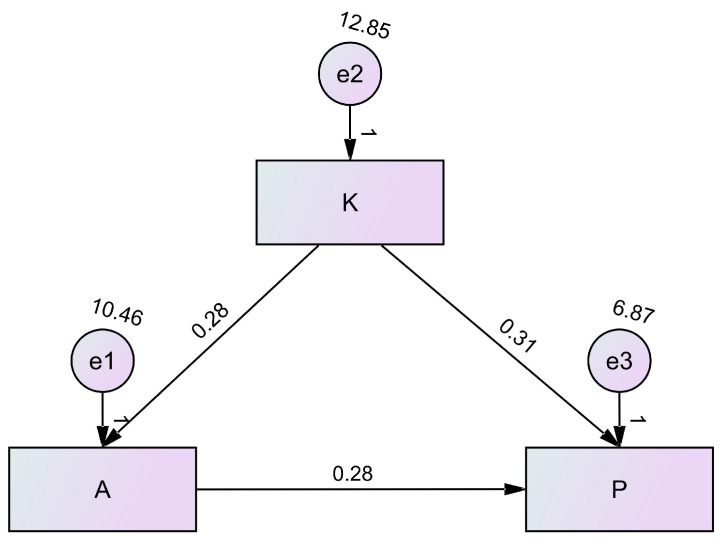
The structural equation model.

Pearson correlation analysis revealed that the knowledge scores of participants were positively correlated with their attitude scores (r = 0.300, *p* < 0.001) and their practice scores (r = 0.450, *p* < 0.001), and their attitude scores were also positively correlated with their practice scores (r = 0.414, *p* < 0.001) (Table 2). The SEM showed that knowledge had a direct effect on attitude (β = 0.283, *p* < 0.001), and attitude had a direct effect on practice (β = 0.280, *p* < 0.001), demonstrating that knowledge had an indirect effect on practice and supporting the first (H1) and third (H3) hypotheses. Knowledge also had a direct effect on practice (β = 0.310, *p* < 0.001) (Table 3 and Figure 3), supporting the second hypothesis (H2).

## 4. Discussion

This study revealed that the dentists in Jiangsu province had overall sufficient knowledge, a positive attitude, and proactive practice with regard to digital dental technologies. The SEM supported the hypotheses made based on KAP theory. These findings may assist in promoting the acceptance and application of digital technologies in dentistry and provide theoretical data to inform future research and quality improvement initiatives.

This study revealed the overall good KAP of dentists regarding digital dental technologies in China. It was reported that the knowledge and experience of dentists could influence their clinical practice behaviors [26], and the results of the current study supported the three hypotheses H1–H3 and validated the causal relationship between KAP dimensions, demonstrating that good knowledge of dentists could improve their attitude and practice with regard to digital dental technologies, and that a positive attitude could also promote the practice of using these technologies. This study is the first to evaluate the relationships between dentists’ KAP dimensions, suggesting that policies should encourage dentists to integrate digital technologies into practice by establishing a sufficient understanding of and positive attitudes towards them. The current study also revealed that gender, age, education, professional title, grade of medical institution, and working experience could influence the KAP of dentists. The positive impact of older age, higher professional titles, higher grades of medical institutions, and more working experience on enhanced KAP is understandable, as dentists can increase their knowledge, skill, attitude, and practice through study and clinical work. A recent study in China has obtained similar findings, whereby respondents with a PhD degree and more years of dental practice used a digital technique (3D-printed implant surgical guides) more frequently [27]. Hence, it is important to improve the overall academic degrees of dentists and provide sufficient targeted training for younger dentists with limited working experience. It was also found that male dentists had significantly higher knowledge and practice scores than females, which requires validation in further studies.

Limited studies have assessed the knowledge of dentists regarding digital dental technologies so far. A total of 96.70% of the participants in this study were aware of CBCT, which was similar to an Indian study that reported that 91.0% of dentists knew about CBCT [28]. A total of 82.55% of the participants in this study knew about 3D printing in dentistry, lower than a study on Arabian dentists (98%) and Indian dental practitioners (90.72%) [29,30]. Generally, socioeconomic status is an important influencing factor associated with KAP [31]. The difference in dentists’ knowledge of digital technologies may be explained by the more developed economic status of Arabia. China and India have similar socioeconomic statuses, and the heterogeneous study population may partially explain their discrepancy in awareness. The knowledge of dentists regarding other technologies, such as intraoral scanning, digital design, CAD/CAM, and surgical guides, has not been reported before; hence, the awareness assessment of dentists regarding these techniques in this study shall provide novel theoretical evidence to inform future research. Despite the overall sufficient knowledge, there are still knowledge gaps in the application of digital technology and techniques including virtual occlusal bracket, medical robotics, and optical navigation in periodontics in China, calling for relevant training programs.

In this study, dentists favored the use of digital dental technologies, with over 90% agreeing that the data of digital technologies is easy to share/store/transfer and that digital technologies can improve working efficiency and enhance patients’ consultation experience. These findings highlighted the advantages of digital dental technologies. Similar positive attitudes have been reported in other studies among dentists and dental students [20,32,33]. In addition, in the present study, most Chinese dentists agreed with the necessity of including the applications of digital technologies in the curriculum of dental students. A national study in Saudi Arabia showed that digital components still need to be integrated into dental schools’ curricula and patient care treatment [34]. A previous randomized controlled trial also revealed that adding digital impression techniques to the dental curriculum could help dental students catch up with ongoing developments in computer-assisted technologies used in dentistry [35]. The findings of this study indicate that education is a promising way to aid with the successful integration of digital technology into future dental practices.

For the practice, dentists in Jiangsu, China, reported overall proactive practices with regard to digital technologies and common applications of CBCT, intraoral scanning, impression scanning, and CAD/CAM, while the necessity of using medical robotics, optical navigation, facial scanning, and virtual occlusal brackets is still controversial. In addition, limited access to and unmet need for virtual occlusal brackets, optical navigation, 3D printing, medical robotics, and surgical guides were found. These findings provided recent data about the application of multiple digital dental technologies in China and filled the research gap. The reasons dentists in this study doubted the necessity of these digital techniques have not been explored. The cost of digital technologies may be a large barrier to the application of digital technologies, as suggested by Jacox et al. [36]. Further studies are still needed to explore these barriers to promote the implementation of digital technologies in dentistry.

There are several limitations in this study. The first limitation is that this study was based on self-reported questionnaire data. Social desirability bias in the participant responses might exist. The second limitation is the selection bias characteristics of survey research. The questionnaires were distributed to dentists through a convenience sampling method through the Jiangsu Dental Association, in which dentists were from different grades of working institutions, and many factors can lead to biased results. To prevent recruiting most dentists from the same hospital or just from large high-grade hospitals, a sample size restriction of 10 dentists from the same medical center was applied. However, the convenience sampling method and the restricted sample size may still have affected the generalization of the findings. Furthermore, the response rate in this study could not be determined due to the online distribution method, which may have influenced the reliability and generalizability of the findings. The third limitation is that this study was a survey conducted in a single province in China, and the socioeconomic status in this region may affect the application of digital technologies in dentistry, which also may affect the generalization of the findings. Another limitation is that although the questionnaire was tested and validated before use, it may not be considered a standardized tool. Further studies with larger samples are needed, possibly via a multi-national study with international standardized questionnaires, to obtain a more precise representation of KAP regarding digital dental technologies among international dentists.

## 5. Conclusions

In conclusion, dentists in Jiangsu province had overall sufficient knowledge, a positive attitude, and good practice with regard to digital dental technologies. Good knowledge of dentists could contribute to better attitudes and practice with regard to digital dental technologies, and a positive attitude could also promote the practice of these technologies. The application of CBCT, intraoral scanning, impression scanning, and CAD/CAM was common, while the necessity of using medical robotics, optical navigation, facial scanning, and virtual occlusal brackets is still controversial. To further improve the acceptance and implementation of digital dental technologies, it might be necessary to enhance education and training for dental practitioners, and policies should encourage dentists to integrate digital technologies into their practice by establishing sufficient understanding and positive attitudes towards them.

## Figures and Tables

**Table 1:** Baseline characteristics of the study population and their KAP regarding digital dental technologies.

Variables	N (%)	Knowledge		Attitude		Practice	
		Mean ± SD	*p*	Mean ± SD	*p*	Mean ± SD	*p*
Total	212	14.33 ± 3.59		26.17 ± 3.40		10.73 ± 3.11	
Gender			0.002		0.610		0.005
Male	108 (50.94)	15.06 ± 3.17		26.29 ± 3.19		11.31 ± 2.82	
Female	104 (49.06)	13.57 ± 3.86		26.05 ± 3.61		10.12 ± 3.29	
Age, years			0.052		0.617		0.010
<40	116 (54.72)	13.79 ± 3.74		26.00 ± 3.46		10.21 ± 3.26 ^#^	
41–50	78 (36.79)	14.91 ± 3.35		26.31 ± 3.50		11.14 ± 2.92	
>50	18 (8.49)	15.28 ± 3.25		26.67 ± 2.52		12.28 ± 2.16	
Professional title			<0.001		0.009		0.007
None	3 (1.42)	10.33 ± 1.53		29.67 ± 2.52		12.67 ± 2.89	
Junior	39 (18.40)	12.64 ± 4.00 ^†^		24.74 ± 4.02 ^†^		9.44 ± 3.26 ^†^	
Intermediate	81 (38.21)	13.98 ± 3.54 ^†^		26.27 ± 3.31		10.56 ± 3.26	
Senior	89 (41.98)	15.53 ± 3.05		26.58 ± 3.01		11.38 ± 2.73	
Ethnicity			0.426		0.167		0.860
Han	208 (98.11)	14.30 ± 3.60		26.13 ± 3.39		10.72 ± 3.09	
Minority	4 (1.89)	15.75 ± 3.20		28.50 ± 3.70		11.00 ± 4.55	
Education			<0.001		0.070		0.003
Bachelor’s degree and below	105 (49.53)	13.45 ± 3.91		25.74 ± 3.61		10.09 ± 3.28	
Master’s degree and above	107 (50.47)	15.20 ± 3.04		26.59 ± 3.14		11.36 ± 2.81	
Subdiscipline			0.152		0.507		0.301
General Dentistry	122 (57.55)	14.08 ± 3.58		26.07 ± 3.22		10.61 ± 3.11	
Endodontics	8 (3.77)	14.25 ± 3.37		24.38 ± 4.72		10.00 ± 2.73	
Prosthodontics	27 (12.74)	15.19 ± 3.76		26.81 ± 3.26		10.37 ± 3.28	
Maxillofacial Surgery	17 (8.02)	15.24 ± 3.46		26.65 ± 2.87		11.53 ± 2.53	
Orthodontics	16 (7.55)	15.06 ± 2.67		26.75 ± 2.82		11.37 ± 3.24	
Oral Implantology	14 (6.60)	14.64 ± 3.88		26.36 ± 4.88		12.00 ± 3.28	
Other	8 (3.77)	11.38 ± 4.03		24.88 ± 4.22		9.13 ± 3.18	
Grade of working institution			0.001		0.477		0.452
Public primary hospital	6 (2.83)	15.83 ± 4.54 ∗		26.00 ± 2.76		10.50 ± 3.39	
Public secondary hospital	25 (11.79)	12.44 ± 3.98 ^∍^		25.96 ± 3.28		10.04 ± 2.44	
Public tertiary hospital	98 (46.23)	14.94 ± 3.36 ∗		26.41 ± 3.09		10.74 ± 2.98	
Private hospital	36 (16.98)	14.69 ± 3.15 ∗		25.58 ± 4.12		11.00 ± 3.02	
Dental clinic	43 (20.28)	14.02 ± 3.52		26.00 ± 3.55		11.12 ± 3.43	
Other	4 (1.89)	9.00 ± 2.45		29.00 ± 3.46		8.25 ± 6.45	
Type of working institution			0.300		0.609		0.175
General hospital	97 (45.75)	14.29 ± 3.43		26.12 ± 3.21		10.34 ± 3.00	
Specialist hospital	61 (28.77)	14.87 ± 3.88		26.33 ± 3.37		11.03 ± 2.85	
Community health service center	2 (0.94)	10.00		23.00 ± 1.41		7.00	
Outpatient department	37 (17.45)	13.95 ± 3.62		25.92 ± 3.77		11.27 ± 3.56	
Other	15 (7.08)	13.93 ± 3.35		26.87 ± 4.02		11.13 ± 3.54	
Work experience, years			0.011		0.344		0.005
<5	28 (13.21)	12.89 ± 3.42 ^∧^		25.54 ± 3.79		10.04 ± 2.67	
5–10	34 (16.04)	13.47 ± 4.15		25.74 ± 3.47		9.41 ± 3.61 ^∧^	
>10	150 (70.75)	14.79 ± 3.40		26.39 ± 3.31		11.15 ± 2.98	

For post hoc pairwise comparisons, ^#^ indicates *p* < 0.05 compared to age > 50 years; ^†^ indicates *p* < 0.05 compared to professional title of senior; ∗ indicates *p* < 0.05 compared to other grade of working institution; ^∍^ indicates *p* < 0.05 compared to public tertiary hospital; ^∧^ indicates *p* < 0.05 compared to work experience of >10 years.

**Table 2:** Correlation analysis between knowledge, attitude, and practice regarding digital dental technologies.

	Knowledge	Attitude	Practice
Knowledge	1		
Attitude	0.300 (*p* < 0.001)	1	
Practice	0.450 (*p* < 0.001)	0.414 (*p* < 0.001)	1

**Table 3:** The structural equation model.

Pathway			Estimate	*p*
H1: Attitude	<---	Knowledge	0.283	<0.001
H2: Practice	<---	Knowledge	0.310	<0.001
H3: Practice	<---	Attitude	0.280	<0.001

## Data Availability

All data generated or analyzed during this study are included in this published article.

## Supplementary Materials

The following supporting information can be downloaded at: https://www.mdpi.com/article/10.3390/healthcare13030234/s1, Figure S1. Confirmatory factor analysis of structural equation model. Table S1. The fitting index of structural equation model.

## Appendix A

**Table A1:** Knowledge.

Knowledge	N (%)		
	Comprehend	Not Comprehend	Uncertain
K2. Are you aware of/Do you understand the digital technologies in dentistry in the following areas?			
Diagnosis	185 (87.26)	11 (5.19)	16 (7.55)
Endodontics	156 (73.56)	35 (16.51)	21 (9.91)
Prosthodontics	194 (91.51)	10 (4.72)	8 (3.77)
Oral Implantology	197 (92.92)	10 (4.72)	5 (2.36)
Orthodontics	177 (83.49)	21 (9.91)	14 (6.60)
Maxillofacial Surgery	138 (65.09)	50 (23.58)	24 (11.32)
Periodontics	95 (44.81)	86 (40.57)	31 (14.62)
K3. Are you aware of/Do you understand the following digital applications in dentistry?			
Cone beam CT (CBCT)	205 (96.70)	3 (1.42)	4 (1.89)
Intraoral scanning/models, digital impression scanning/facial scanning	198 (93.40)	9 (4.25)	5 (2.36)
CAD/CAM	177 (83.49)	21 (9.91)	14 (6.60)
3D printing	175 (82.55)	24 (11.32)	13 (6.13)
Virtual occlusal bracket	95 (44.81)	87 (41.04)	30 (14.15)
Surgical guides	175 (82.55)	24 (11.32)	13 (6.13)
Optical navigation	86 (40.57)	91 (42.92)	35 (16.51)
Medical robotics	94 (44.34)	86 (40.57)	32 (15.09)
K4. The results obtained using digital scanning are more accurate than traditional impressions.	150 (70.75)	8 (3.77)	54 (25.47)
K5. Digitally designed and produced restorations give better outcomes than traditional methods.	144 (67.92)	6 (2.83)	62 (29.25)
K6. Scanning and modeling using digital technologies allow for real-time modifications and feedback.	190 (89.62)	2 (0.94)	20 (9.34)
K7. The data of digital technologies is easy to share/store/transfer.	207 (97.64)	0	5 (2.36)

**Table A2:** Attitude.

Attitude	N (%)				
	Strongly Agree	Agree	Neutral	Disagree	Strongly Disagree
A1. Digital technologies can improve the efficiency of dentists.	131 (61.79)	66 (31.13)	13 (6.13)	2 (0.94)	0
A2. Digital technologies can enhance the consultation experience of dental patients.	130 (61.32)	71 (33.49)	10 (4.72)	1 (0.47)	0
A3. The widespread use of digital technologies is a developing trend in dentistry.	146 (68.87)	62 (29.25)	4 (1.89)	0	0
A4. Digital technologies should be introduced to patients before use.	116 (54.72)	84 (39.62)	10 (4.72)	1 (0.47)	1 (0.47)
A5. Digital technologies play an irreplaceable role in the diagnosis and treatment of dentistry.	69 (32.55)	58 (27.36)	58 (27.36)	27 (12.74)	0
A6. The applications of digital technologies in dentistry still rely on professional dentists.	123 (58.02)	71 (33.49)	13 (6.13)	4 (1.89)	1 (0.47)
A7. The applications of digital technologies should be included in the curriculum of dental students.	122 (57.55)	74 (34.91)	16 (7.55)	0	0
A8. I am concerned that I may be replaced by digital technologies in the future.	20 (9.43)	25 (11.79)	80 (37.74)	74 (34.91)	13 (6.13)

**Table A3:** Practice.

Practice	N (%)				
	Always	Often	Sometimes	Occasionally	Never
P1. The frequency I recommend patients to undergo digital imaging	47 (22.17)	90 (42.45)	50 (23.58)	22 (10.38)	3 (1.42)
P2. The frequency I use digital technologies to assist in the treatment of dental diseases	37 (17.45)	93 (43.87)	47 (22.17)	27 (12.74)	8 (3.77)
P3. The frequency I introduce the use of digital technologies to patients	55 (25.94)	88 (41.51)	46 (21.70)	20 (9.43)	3 (1.42)
P4. The frequency I proactively learn about advances in the application of digital technologies	36 (16.98)	81 (38.21)	72 (33.96)	19 (8.96)	4 (1.89)
