# Supplementary material for: Knowledge, Attitude, and Practice Regarding Digital Dental Technologies Among Dentists in Jiangsu Province
Source: Healthcare (Basel). 2025 Jan 24;13(3):234. doi: 10.3390/healthcare13030234 (PMC11817746; doi:10.3390/healthcare13030234)
Supplement: Supplementary file 1 [file healthcare-13-00234-s001.zip › healthcare-3343642-supplementary.pdf]

Supplementary Figure S1. Confirmatory factor analysis of structural equation model

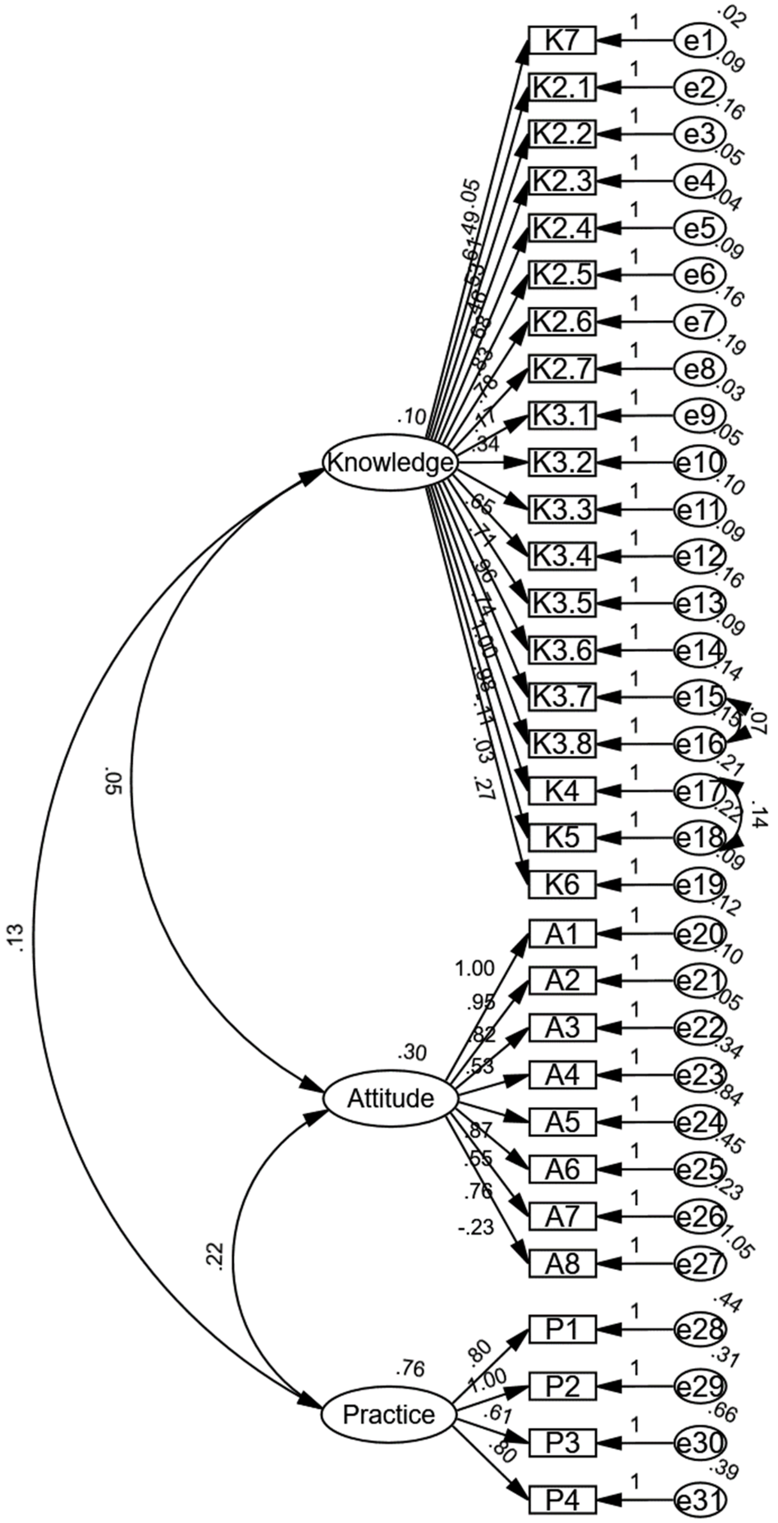

Supplementary Table S1. The fitting index of structural equation model

Indicators	Reference	Actual
CMIN/DF	1-3: Excellent, 3-5: Good	1.910
RMSEA	<0.08: Good	0.066
IFI	>0.8: Good	0.828
TLI	>0.8: Good	0.811
CFI	>0.8: Good	0.825
